# Homology-Driven Proteomics of Dinoflagellates with Unsequenced Genomes Using MALDI-TOF/TOF and Automated *De Novo* Sequencing

**DOI:** 10.1155/2011/471020

**Published:** 2011-09-29

**Authors:** Da-Zhi Wang, Cheng Li, Zhang-Xian Xie, Hong-Po Dong, Lin Lin, Hua-Sheng Hong

**Affiliations:** State Key Laboratory of Marine Environmental Science/Environmental Science Research Center, Xiamen University, Xiamen 361005, China

## Abstract

This study developed a multilayered, gel-based, and underivatized strategy for *de novo* protein sequence analysis of unsequenced dinoflagellates using a MALDI-TOF/TOF mass spectrometer with the assistance of DeNovo Explorer software. MASCOT was applied as the first layer screen to identify either known or unknown proteins sharing identical peptides presented in a database. Once the confident identifications were removed after searching against the NCBInr database, the remainder was searched against the dinoflagellate expressed sequence tag database. In the last layer, those borderline and nonconfident hits were further subjected to *de novo* interpretation using DeNovo Explorer software. The *de novo* sequences passing a reliability filter were subsequently submitted to nonredundant MS-BLAST search. Using this layer identification method, 216 protein spots representing 158 unique proteins out of 220 selected protein spots from *Alexandrium tamarense*, a dinoflagellate with unsequenced genome, were confidently or tentatively identified by database searching. These proteins were involved in various intracellular physiological activities. This study is the first effort to develop a completely automated approach to identify proteins from unsequenced dinoflagellate databases and establishes a preliminary protein database for various physiological studies of dinoflagellates in the future.

## 1. Introduction

Dinoflagellates are a diverse group of unicellular algae that comprise a large part of the marine and freshwater phytoplankton [1]. They are not only the important primary producers and an important part of the food chain in marine ecosystem, but also the major causative species resulting in harmful algal blooms (HABs) in the coastal zone [2]. Moreover, many dinoflagellate species can produce various potent toxins that impact human health through the consumption of contaminated shellfish, through coral reef fish and finfish, or through water or aerosol exposure [3]. At the present, four major seafood poisoning syndromes caused by toxins have been identified from the dinoflagellates: paralytic shellfish poisoning (PSP), diarrheic shellfish poisoning, neurotoxic shellfish poisoning, and ciguatera fish poisoning. It is estimated that dinoflagellate toxins result in more than 50,000–500,000 intoxication incidents per year, with an overall mortality rate of 1.5% on a global basis [4]. In addition to their adverse effects on human health, dinoflagellate toxins are responsible for the death of fish and shellfish and have caused episodic mortalities of marine mammals, birds, and other animals dependant on the marine food web [5–8]. Dinoflagellate causing HABs and toxin-producing dinoflagellates have become a global concern [3, 9, 10].

Dinoflagellates are notable for their unusual genome content and organization [11, 12]. It is estimated that the dinoflagellate DNA content ranges from 3 to 250 pg·cell^−1^ [13, 14], corresponding to approximately 3,000–215,000 Mb. Moreover, dinoflagellates have many chromosomes (up to 325) that are permanently condensed and attached to the nuclear envelope during cell division. These unique features of dinoflagellates have brought challenges to the use of traditional biochemical methods and molecular technology in the study of dinoflagellates [15], and so genetic information concerning dinoflagellates are lacking worldwide at present, which has seriously impeded our understanding of HABs and, consequently, the monitoring, mitigation, and prevention. 

Proteins are the actual “machinery” that brings about cell growth, proliferation, and homeostasis, and it is logical, therefore, that the study of proteins should help uncover in broad terms the various mechanisms involved in the biological activities of dinoflagellates. Global techniques such as proteomics provide effective strategies and tools for profiling and identifying dinoflagellate proteins, and, in contrast to conventional biochemical approaches that addressed one or a few specific proteins at a time, the proteomic techniques allow simultaneous isolation and identification of hundreds to thousands of proteins in one sample. In the past few years, the proteomic approach has been applied to the study of dinoflagellates, and a few important proteins have been discovered or identified [16–18]. However, only 3,578 and 2,621 dinoflagellate proteins are annotated in the NCBI and UniProtKB (December, 2010), respectively. The highly uncharacterized nature of the dinoflagellate proteome makes it difficult to identify proteins, demonstrate differential regulation of proteins, and investigate their posttranslational modifications. The lack of a genome limits the use of dinoflagellates for proteomic studies which rely on database searches for protein identification. Recently, with the fast development of MALDI-TOF-TOF MS technology, this limitation has been overcome to some extent using a *de novo* sequencing strategy, in which partial or complete amino acid sequences are obtained using either manual or automated *de novo* peptide sequence analysis. This approach has been successfully applied in recent studies with incomplete- or nongenome organisms in order to characterize their proteins [19–23].

*Alexandrium* is a widely distributed dinoflagellate genus in many coastal regions around the world. It is well known that many species from this genus can produce potent neurotoxins which cause PSPs through the consumption of shellfish contaminated by toxins [24, 25]. The losses in mariculture and the threats to human life due to exposure to PSPs have been documented increasingly and have become economic and public health concerns around the world. Recently, many efforts have been devoted to establish the expressed sequence tag (EST) library of *Alexandrium* and other dinoflagellate species, which provides a powerful tool to predict protein families and to develop expression systems for new proteins and their functions [26–28]. Our study selected *A. tamarense* as the model dinoflagellate species, and a layered method combining MALDI-TOF-TOF MS with *de novo* sequence analysis and stringent homology-based searching tools was employed to identify the proteins. A highly specific and stringent MASCOT search was applied as the first layer to identify proteins with identical peptides in the present database; the remainder were searched against a dinoflagellate EST database combined with BLASTX analysis. In the last layer, those borderline and nonconfident hits were subjected to automated* de novo *sequencing and homology searches using the homology-based search algorithm, MS-BLAST. Using this strategy, 158 unique proteins in 220 selected protein spots were identified from *A. tamarense*, and these proteins were involved in various physiological activities. The current study validated a robust method to characterize proteins from an unsequenced database of *A. tamarense *thereby facilitating the use of this HAB model in various studies.

## 2. Materials and Methods

### 2.1. Organism and Growth Conditions

The strain of *A. tamarense* was provided by the Culture Collection Center of Marine Bacteria and Algae of the State Key Laboratory of Marine Environmental Science, Xiamen University, China. The unialgal isolate was routinely maintained in K medium [29] at 20°C under a 14 : 10 h light : dark photoperiod at a light intensity of approximately 100 *μ*moL photons m^−2^ s^−1^ provided by fluorescent lamps. The cells for the experiments were grown in 5,000 mL flasks containing 4,000 mL of K medium, the culture conditions were the same as above. The K-medium did not contain any protein. Approximately 2 × 10^7^  cells of *A. tamarense *in the middle exponential growth phase were collected by centrifugation at 3,000 ×g for 30 minutes at 4°C. The cell pellets were rinsed twice with precooled sterilized seawater to avoid any carryover of culture medium and extracellular proteins, ready for protein extraction.

### 2.2. Protein Extraction and Determination

Protein extraction was performed according to the method developed by Lee and Lo [30]. Briefly, 1 mL Trizol reagent was added to the cell pellet and subjected to sonication (a total of 2 min with short pulses of 3–5 s) on ice. Lysis of cells was confirmed using light microscope. Subsequently, 200 *μ*L of chloroform was added to the cell lysate before shaking vigorously for 15 s. The mixture was allowed to stand for 5 min at room temperature before being centrifuged at 12,000 ×g for 15 min at 4°C The top pale yellow or colorless layer was removed, and then 300 *μ*L of ethanol was added to resuspend the reddish bottom layer, and the mixture centrifuged at 2,000 ×g for 5 min at 4°C. The supernatant was transferred to a new tube, and 2 mL of isopropanol was added. The mixture was allowed to stand for at least 1 hr for precipitation of proteins at −20°C. It was then centrifuged at 14,000 ×g for 30 min at 4°C. The pellet obtained was briefly washed with 95% ethanol before being allowed to air dry. 30 *μ*L of rehydration buffer (7 M urea, 2 M thiourea, 4% w/v CHAPS, 1% DTT, and 0.5% v/v IPG) was added to solubilize the protein pellet. Protein quantification in the urea-containing protein samples was performed using a 2D Quant kit (GE Healthcare, USA). 

### 2.3. 2-DE and Analysis

Exactly 400 *μ*g of protein sample was mixed with a rehydration buffer (7 M urea, 2 M thiourea, 4% w/v CHAPS, 1% DTT, and 0.5% v/v IPG) before being loaded onto IPG strips with a linear pH gradient of 4–7 (Immobiline Drystrip, pH 4–7, GE Healthcare Life Science, Piscataway, USA). The sample was subjected to isoelectric focusing using an IPGphor III system with 24 cm IPG strips following the manner: 6 h at 40 V (active rehydration), 6 h at 100 V, 0.5 h at 500 V, 1 h at 1,000 V, 1 h at 2,000 V, 1.5 h at 10,000 V, and 60,000 Vh at 10,000 V. The minimal Vh applied was at least 60,000 units. Subsequently, the immobilized pH gradient strips were equilibrated for 15 min in reducing buffer containing 6 M urea, 2% SDS, 50 mM Tris-Cl (pH 8.8), 30% glycerol, and 1% DTT, followed by equilibration for 15 min in alkylation buffer containing 6 M urea, 2% SDS, 50 mM Tris-Cl (pH 8.8), 30% glycerol, and 2.5% iodoacetamide. Two-dimension SDS-PAGE (2-DE) gels (12.5%) were run in an EttanDalt system (GE Healthcare) at 1 w/gel for 30 min and then at 15 w/gel for 6 h. The 2-DE gels were visualized using Coomassie Blue (CBB) staining and digitized using a gel documentation system on a GS-670 Imaging Densitometer from Bio-Rad (USA) with 2-DE electrophoretogram-matching software. 

### 2.4. In-Gel Trypsin Digestion

Two hundred and twenty protein spots were manually excised from preparative CBB stained 2-DE gels (Figure 2). CBB-stained gel pieces were washed with MilliQ water for 10 min, destained three times in 200 *μ*L of 25 mM NH_4_HCO_3_ in 50% acetonitrile (ACN) for 20 min at 37°C, and then incubated in 200 *μ*L of 100% ACN at room temperature with occasional vortexing, until the gel pieces became white and shrunken. They were then air dried at room temperature for 30 min. All gel pieces were incubated with 12.5 ng/*μ*L sequencing-grade trypsin (Roche Molecular Biochemicals) in 10 mM NH_4_HCO_3_ overnight at 37°C. After digestion, the supernatants were discarded. Peptides were extracted from the gel pieces first into 50% ACN, 0.1% trifluoroacetic acid, and then into 100% ACN. All extracts were pooled and dried completely by SpeedVac. Peptide mixtures were redissolved in 0.1% TFA, and 1 *μ*L of peptide solution was mixed with 1 *μ*L of matrix (*α*-cyano-4-hydroxycinnamic acid (CHCA) in 30% ACN, 0.1% TFA) before spotting on the target plate.

### 2.5. Mass Spectrometric Analysis

Mass spectrometry analyses were conducted using an AB SCIEX MALDI TOF-TOF 5800 Analyzer (AB SCIEX, Shanghai, China) equipped with a neodymium: yttrium-aluminum-garnet laser (laser wavelength was 349 nm), in reflection positive-ion mode. With CHCA as the matrix, TFA for an ionization auxiliary reagent, and calibrated with Sequenzyme peptide standard kit (AB SCIEX), the MS spectra were processed using TOF/TOF Series Explorer software (AB SCIEX) allowing nonredundant and fully automated selection of precursors for MS/MS acquisition. At least 1,000 laser shots were typically accumulated with a laser pulse rate of 400 Hz in the MS mode, whereas in the MS/MS mode spectra up to 2,000 laser shots were acquired and averaged with a pulse rate of 1,000 Hz. Peptides were fragmented with collision-induced decomposition (CID) with an energy of 1 kV. For CID experiments, ambient air was used as collision gas with medium pressure of 10^−6^ Torr. The 20 most intense precursors per spot were selected with a minimum signal-to-noise (*S/N*) ratio of 50 and were fragmented in the CID mode. The peak detection criteria used were a minimum *S/N* of 10, a local noise window width mass/charge (*m*/*z*) of 200 and a minimum full-width half-maximum (bins) of 2.9. The contaminant *m/z* peaks originating from human keratin, trypsin autodigestion, or matrix were included in the exclusion list used to generate the peptide mass list for the database search.

### 2.6. *De Novo* Sequencing

The Applied Biosystem DeNovo Explorer software (AB SCIEX) was used for automated *de novo *sequencing followed by manual confirmation of most sequences generated. Those nonconfident fits were submitted to *de novo* sequencing analysis. The *de novo *sequencing parameters were set as follows: trypsin as the protease with one maximum missed cleavage allowed, the error tolerance of a parent- and fragment-mass was 0.08 u, deconvolute the charge state in the spectra to generate a spectrum in which each monoisotopic peak becomes singly charged, carbamidomethylation of cysteine as fixed modification and methionine oxidation as variable modification. The most abundant peptide fragments “*b-ions *and *y-ions*”, the less abundant peptide fragments “*a-ions*”, the neutral losses of water and ammonia for *b-ions *and *y-ions*, as well as the *immonium ions *were used to deduce confident and complete peptide sequences *de novo *from MS/MS spectra. Each MS/MS spectrum produced ten peptide sequence candidates, and each peptide sequence had a score associated with it that indicated how much of the total ion abundance in the MS/MS spectrum was accounted for by the typical fragment ions that can be calculated for the particular sequence; the closer the score was to 100, the greater the likelihood that all or most of the sequence generated by the DeNovo Explorer was corrected. In order to minimize randomness, only those peptides with a score higher than 50 were considered in this study. 

### 2.7. Database Searches

A combined MS and MS/MS search was first performed against the NCBI database with no taxonomic restriction (updated December, 2010, containing 4,607,655 entries) using an in-house MASCOT server (Version 2.2). The raw MS and MS/MS spectra were processed using GPS Explorer software (Version 3.5, Applied Biosystems, USA) with the following criteria: MS peak filtering mass range, 850–4,000 Da; minimum signal-to-noise ratio, 10; peak density filter, 50 peaks per 200 Da; maximum number of peaks, 65; MS/MS peak filtering-mass range, 60–200 Da. The searches were conducted using the following setting: one missed cleavage, *P* < 0.05 significance threshold, 50 ppm peptide mass tolerance, 0.25 Da fragment mass tolerance peptide mass tolerance of 50 ppm, MS/MS ion tolerance of 0.1 Da, carbamidomethylation of cysteine as fixed modification, and methionine oxidation as variable modification. For a protein scores confidence interval (C.I.) below 95%, the MS/MS spectra were subjected to similarity searches against the dinoflagellate EST database (downloaded from NCBI, updated December, 2010, containing 171,550 entries) using the BLASTX algorithm [31]. The similarities were considered to be significant when the total ion C.I. % was ≥95, and the *E* value was below e^−20^. Nonetheless, the remaining hits were further identified using *de novo *sequencing and homology-based search as previously described [32]. 

*De novo *generated peptide sequences were used for homology searches using the MS BLAST algorithm. The MS-BLAST searches were conducted via the Washington University server (http://genetics.bwh.harvard.edu/msblast/disclaimer_ms.html) against the NCBI nonredundant database using standard settings with no taxonomic restriction. All sequences obtained from a MS/MS spectrum were spaced with the minus symbol (−) and were merged into a single string and submitted to an MS-BLAST search as reported before [33, 34]. The MS-BLAST search results were regarded as significant if the resulting scores were higher than the threshold score indicated in the MS-BLAST scoring scheme. However, only high-scoring segment pairs (HSSPs) with a score of 62 or above were considered. The clusters of orthologous groups [35] databases were used to infer the functional classification of the proteins identified. 

## 3. Results

### 3.1. The Workflow of Protein Identification

**Figure 1:**
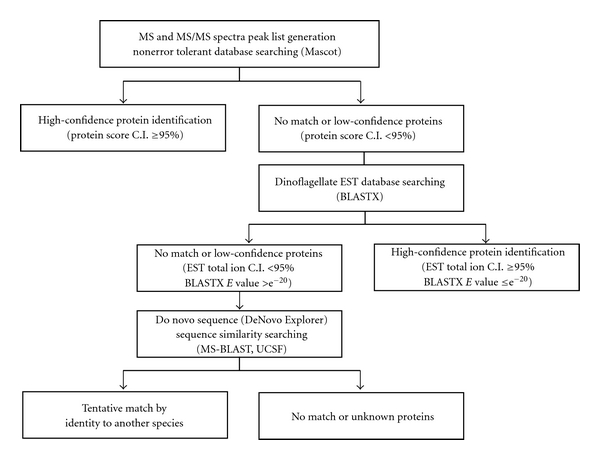
Multilayered protein identification workflow. After MASCOT search against the NCBI database, confident hits were identified with at least two peptides and protein scores above the minimum C.I. of 95%. Cross-species hits matching one peptide or protein scores below C.I. 95% were considered as borderline and were subjected to similarity searches against the dinoflagellate EST database using the BLASTx algorithm. The sequence similarities were considered to be significant if total ions score C.I. was ≥95% and the *E* value was ≤e^−20^ at the amino acid sequence level. Nonconfident hits were interpreted using DeNovo Explorer software and MS-BLAST searches. Only HSPs with a score of 62 or above were considered confident.

The multilayered workflow integrated mass spectra processing with conventional and homology-based searches is outlined in Figure 1. Briefly, the MS and MS/MS spectra of each protein spot obtained from MALDI-TOF-TOF MS were first submitted to MASCOT search against the NCBI database with no taxonomic restriction. If the database entries were matched with at least two peptides and the protein scores taken from MS combined MS/MS search had a minimum C.I. of 95%, the protein hits were regarded as confident identifications. Cross-species hits matching one peptide or protein scores below a C.I. of 95% were considered as low-confidence identifications, and the MS/MS spectra were subjected to similarity searches against the dinoflagellate EST database. The sequences were then subjected to similarity searches against the NCBI nonredundant protein database (nr) using the BLASTX algorithm [31]. If the total ions score C.I. was above 95% and the *E* value was below e^−20^ at the amino acid sequence level, the sequence similarities were considered to be significant. In the last layer, those nonconfident hits were sequenced using *de novo* sequencing software to obtain candidate sequences and submitted to MS-BLAST searches. In the homology-based search, the statistical significance of hits was evaluated according to the MS BLAST scoring scheme. Only HSSPs with a score of 62 or above were considered to be confident [36, 37].

### 3.2. Protein Identification Using Mascot and Dinoflagellate EST Searches

**Figure 2:**
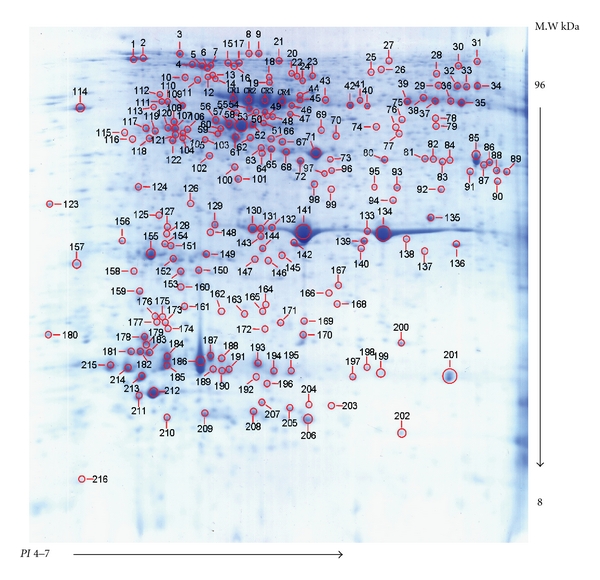
Representative 2-DE gel of an *A. tamarense *protein sample stained with CCB. The proteins were resolved in a linear 4–7 pH gradient (Immobiline DryStrips) and 12.5% SDS-PAGE.

The protein extract from *A. catenella *was separated using 2-DE and visualized using the modified CBB stain method. An average of about 880 protein spots was detected in the 2-DE gel (Figure 2). Among them, 220 representing low, moderate, and high abundance intracellular proteins were randomly excised from the 2-DE gel and were in-gel digested using trypsin after destaining the gel plugs. The peptide fragments extracted from the gel plugs were subjected to tandem mass spectrometry using the AB SCIEX MALDI-TOF/TOF 5800 System. Tandem mass spectra excluding contaminant peaks from human keratin, trypsin autodigestion, or matrix were directly submitted for database searching (GPS Explore: MASCOT) for protein characterization using the NCBInr database with or without all known posttranslational modifications. Out of the 220 protein spots, 104 were identified statistically as cross-species matches yielding positive characterization and high matching score in MASCOT searches and accounted for a half of the totally identified proteins (see Supplemental file 1 available online at doi:10.1155/2011/471020). Among them were 100 protein spots with two or more MS/MS significant hits, and four protein spots with one MS/MS significant hit which was regarded as the borderline. A large proportion of the identified proteins showed a high level of similarity to the proteins of dinoflagellates (49.0%), nondinoflagellate algae (8.7%), and other species of organisms (42.3%) (Figure 5(a)).

The remaining 116 protein spots with low protein scores (<C.I 95%) as well as those proteins with one MS/MS hit were subjected to search against the EST database about dinoflagellate sequences, combining with BLASTX analysis. With a stringent cut-off *E* value of e^−20^ or less and a total ion C.I. % of ≥95, a total of 72 sequence similarities were confidently identified in *A. tamarense* (Supplemental file 1). A large proportion of the identified proteins showed a high level of similarity to dinoflagellate proteins (59.7%), nondinoflagellate algae (11.1%), and other species of organisms (29.2%) (Figure 5(b)). The rest of the protein spots with nonconfident hits were subsequently identified using a combination of *de novo *sequencing and MS-BLAST searches.

### 3.3. Protein Identification Using *De Novo* Sequencing and MS-BLAST Searches

Typically, the 20 most intense peaks in the PMF were selected for MS/MS analysis. The tandem mass spectra were analyzed using DeNovo Explorer software to generate amino acid sequences and deconvoluted to minimize the error in *de novo* sequencing. DeNovo Explorer works in the same way as PEAKS: briefly, the algorithm first computes a *y*-ion matching score and a *b*-ion matching score at each mass value according to the peaks around it. If there are no peaks around a mass value, a penalty value is assigned. The algorithm then efficiently computes many amino acid sequences, and each candidate peptide sequence is assigned a score that indicates the degree of matching of the peaks and the intensity of the peaks between the theoretical fragmentation spectrum and the fragmentation spectrum that corresponds to the peaks in the peak list. The scores in the Denovo Explorer are calculated based on the percent peak intensity match of the fragments between the actual data and the candidate peptide. These candidate sequences are further evaluated by a more accurate scoring function, which also considers other ion types such as *immonium* ions and internal cleavage ions [32]. 

**Figure 3:**
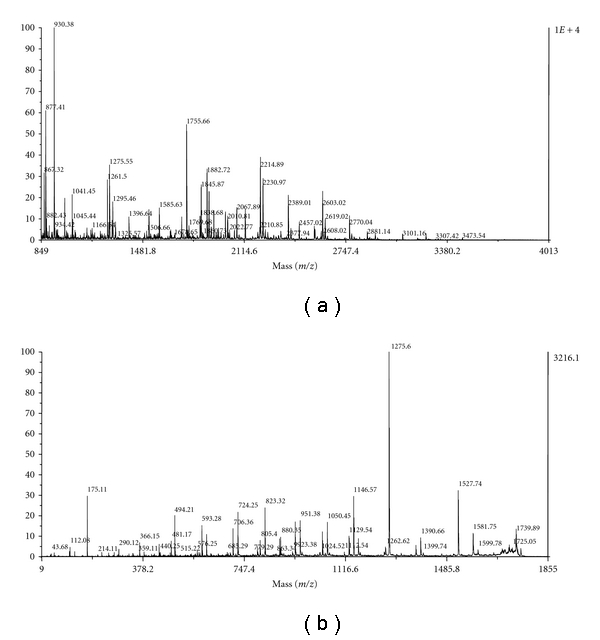
Peptide mass fingerprint and MS/MS spectrum (peptide 1755.6631) derived from spot 124 in Figure 2.

**Figure 4:**
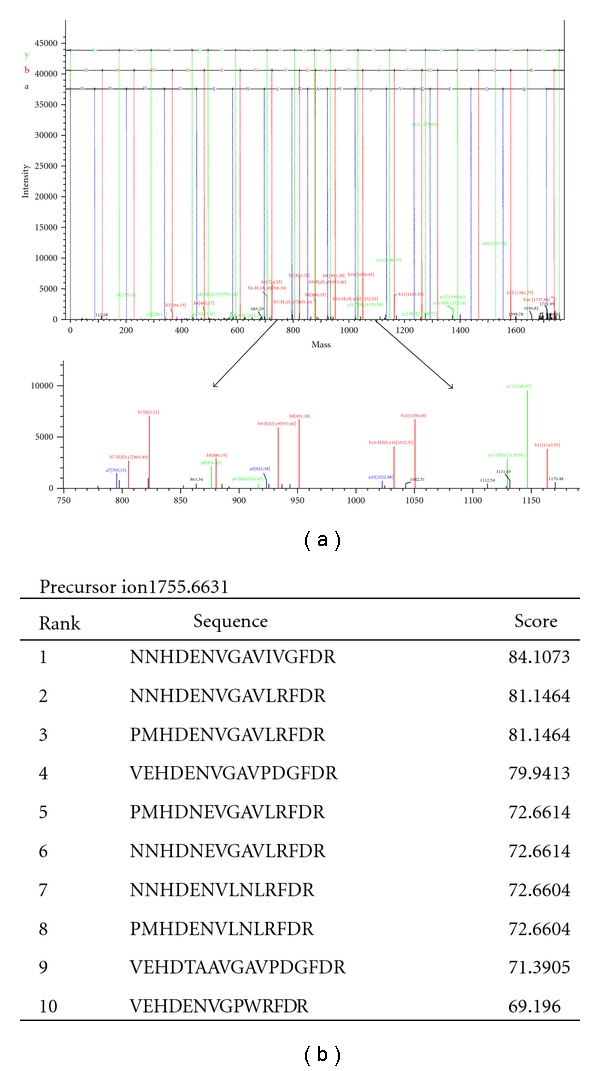
*De novo *analysis of an unknown protein from *A. tamarense*. (a) The *x*- and *y*-axes show the mass-to-charge (*m/z*) ratio and the % abundance of the precursor ion fragments (*m/z* of 1755.6631), respectively. The MS/MS spectrum was analyzed using DeNovo Explorer software to generate “NNHDENVGAVIVGFDR”, and (b) the table details ten peptide sequence candidates for this precursor deduced from DeNovo Explorer *de novo *sequencing.

In most spots, 100 to 200 amino acid sequences, with a length varying between seven and 37 amino acids, were obtained *de novo*. In this study, the *de novo *sequencing selects the most abundant peptide fragments “*b*-*ions*” and “*y*-*ions*”, less abundant peptide fragments “*a*-*ions*”, and the neutral losses of water and ammonia for *b-ions* and *y-ions* as well as *immonium* ions to generate confident peptide sequences *de novo* from MS/MS spectra.  Figure 3 shows the MS spectrum of the in-gel tryptic peptide mixture of spot 124, and displays the fragmentation pattern of a precursor ion with *m/z* of 1755.6631 from spot 124 and the* b*-, *y*-,* a*-, and *immonium ions* as well as the neutral losses of water and ammonia for *y*-*ions *and* b*-*ions* (Figure 4(a)). Ten possible peptide sequences for this precursor were deduced from DeNovo Explorer *de novo *sequencing and are listed in the order according to their scores in Figure 4(b). The peptide sequence candidate with the highest score for this precursor was “NNHDENVGAVIVGFDR” deduced from DeNovo Explorer *de novo *sequencing. A similar analysis was performed on the other selected protein spots.

The *de novo *deduced peptide sequences were used to identify the proteins using sequence similarity searching. Several database searching tools have been developed that accommodate the specific requirements of MS/MS sequencing [27, 38]. In our study, the homology-based data search approach MS-BLAST was used. This is the most popular database search approach for identifying unknown proteins using sequence similarity to homologous proteins available in a database. The redundant, degenerate, and partially inaccurate peptide sequences obtained by *de novo *interpretation of MS/MS spectra are assembled into a single searching string in arbitrary order [33, 37]. The quality of the results is dependent on the number of peptides sequenced and the accuracy of the sequence information entered, as well as database completeness and species-to-species sequence variability for the peptides entered. It is also possible to enter a part of the sequence as a mass, along with a tolerance factor.

**Figure 5:**
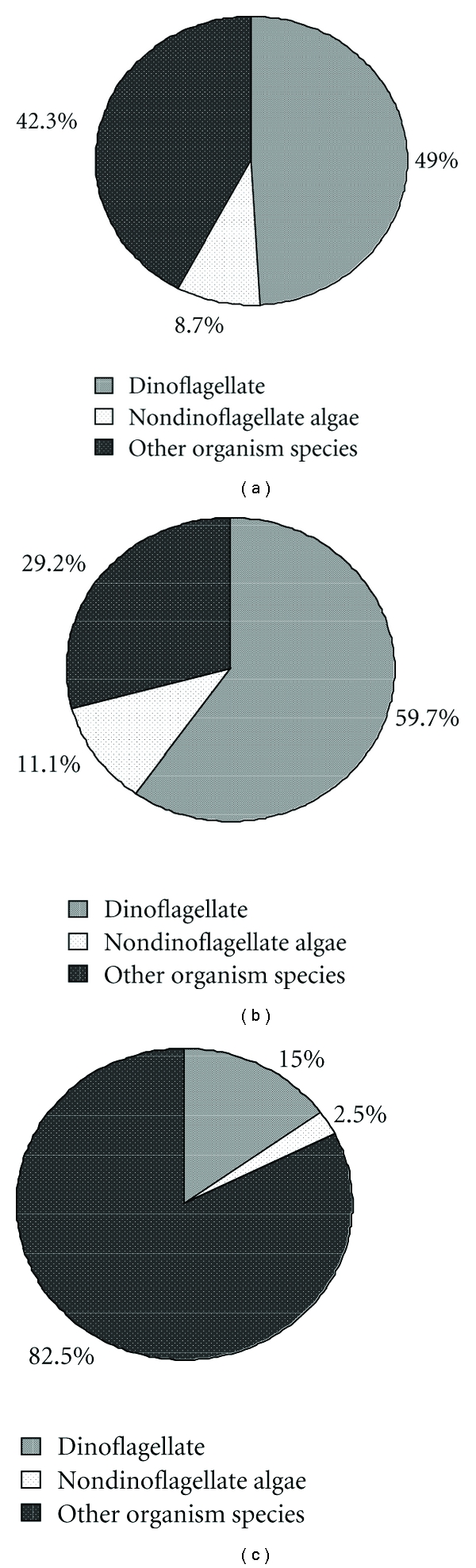
Taxonomic group distribution of proteins from *A. tamarense*. (a) Proteins identified using MASCOT search against the NCBInr database, (b) proteins identified against the dinoflagellate EST database, and (c) proteins identified with *de novo* and MS-BLAST search.

The *de novo *derived sequence information from each protein spot with nonconfident hits was combined in one search query and analyzed using the MS BLAST algorithm. The results were chosen according to the number of HSSPs from different MS/MS spectra [37], and phylogenetic closeness to dinoflagellates was also considered. Using this strategy, 40 protein spots out of 44 protein spots were tentatively identified, 32 of them obtaining two or more HSSP significant hits and eight only one. However, four protein spots could not obtain positive identification and were assigned to unknown proteins (Supplemental file 1). A large proportion of the identified proteins showed a high level of similarity to proteins of dinoflagellates (15.0%), non-dinoflagellate algae (2.5%) and other species of organisms (82.5%) (Figure 5(c)).

### 3.4. Validation of MASCOT Cross-Species Identifications with Borderline Statistical Confidence

Cross-species identification of proteins by matching identical peptides in known homologous proteins is a conventional proteomic methodology. However, such identification often results in borderline statistical confidence due to the relatively rare peptides and only a few peptide sequences matching. Here, we demonstrate how *de novo* sequencing and MS BLAST searches provided independent validation of borderline cross-species MASCOT hits [39]. The MS BLAST scoring scheme and its validation are described elsewhere [37]. 

**Figure 6:**
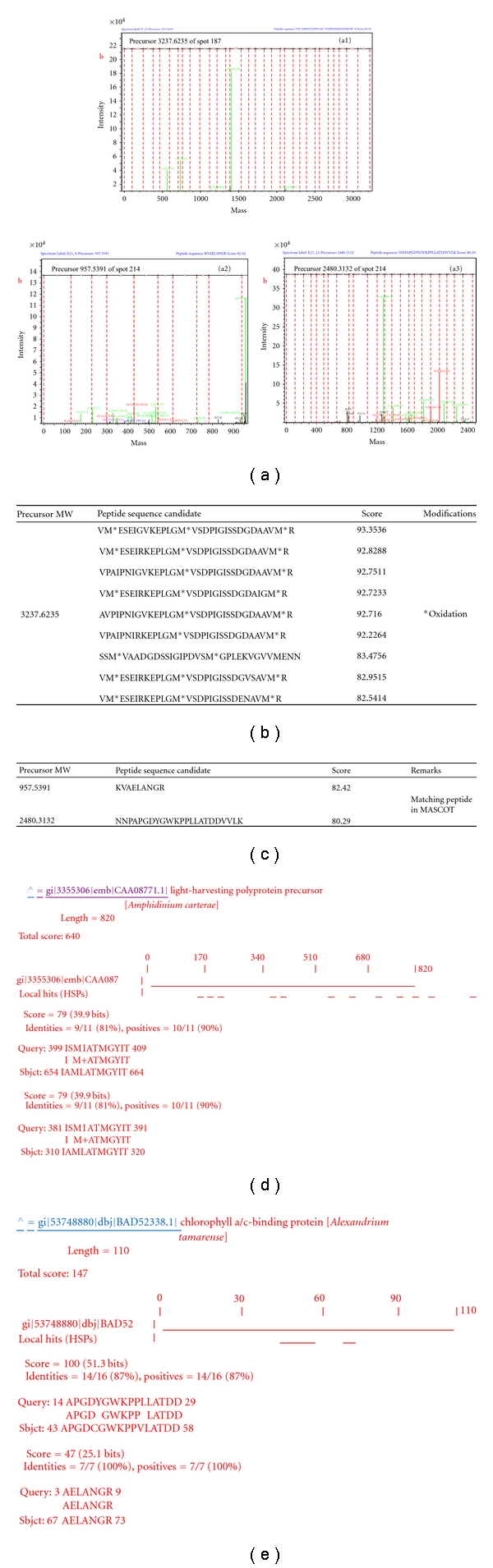
*De novo* sequencing and an MS-BLAST search validated a borderline hit produced using the MASCOT search. (a) The *x*- and *y*-axes show the mass-to-charge (*m/z*) ratio and the % abundance of the precursor ion fragments, respectively. The MS/MS spectrum was analyzed using DeNovo Explorer software to generate peptide (precursor 957.5391, 2480.3132, and 3237.6235) sequence candidates, (b) the table details ten peptide sequence candidates for the precursor 3237.6235 deduced from *de novo* sequencing, (c) the file corresponding to the spectrum in a2 and a3 and their *de novo* interpretation produced two candidate sequences with the quality score, and (d) and (e) the peptide sequence candidates from (b) and (c) were merged into an MS-BLAST query, and the search hit the same protein from *A. tamarense*. According to the MS-BLAST scoring scheme, the hits were confident.

In spot 187 of the above sample of *A. tamarense* proteins, a MASCOT search identified a plausible homologue of the chloroplast light harvesting complex protein from another algal species, *Heterocapsa triquetra*. However, this identification relied upon a single exactly matching peptide, and, in line with current proteomics guidelines [40], it should be considered as borderline. To validate this hit, the MS/MS spectrum was then interpreted *de novo *(Figures 6(a1) and 6(b)), and the top ten candidate sequences were linked in a string and submitted to MS BLAST search (Figure 6(e)), which produced a statistically confident hit from *A. carterae* to the overlapping sequence stretch in a related database entry. It should be noted that peptide sequences of the MASCOT hit and *de novo* candidates differed in their length of amino acid sequence, and, currently, it is not possible to judge which peptide sequence was correct, since the full sequence of the *A. tamarense* protein remains unknown. The two proteins from the MASCOT hit and MS BLAST search were homologous. However, this did not affect the confidence of the MS BLAST hit assignment, which relies upon an independent scoring scheme that only considers the local similarity of sequence stretches aligned within the HSP. In regard to spot 214, the MASCOT hit and the result of MS BLAST search using *de novo* candidates were identical using validated methods [36] (Figures 6(a2), 6(a3), 6(c) and 6(e)). Additionally, MS BLAST searches also revealed one new peptide (precursor MW 2480.3132) from a protein already matched by MASCOT (spot 214) thus improving the sequence coverage and confidence of identification.

### 3.5. Functional Categorization of the Proteins Identified from *A. tamarense*

Using the multilayer, stringent, and homology-similarity database searching strategy, 216 protein spots (representing 158 unique proteins) were identified from *A. tamarense* out of the 220 protein spots isolated. The remaining four protein spots did not give positive identification and were assigned to unknown proteins. The NCBI accession number, protein name, protein score and C.I. %, total ion score and C.I. %, number of unique peptides and total spectra used in the identification; and the theoretical MW and isoelectric point of the proteins identified are listed in the Web Appendix.

It should be pointed out that many of the proteins identified presented multiple isoforms in 2-DE gel with different *PI* and MW values, thus forming a train of spots horizontally or scattering on the 2-DE gel. For example, four isoforms of ribulose-1,5-bisphosphate carboxylase oxygenase (RuBisCO), CR1, CR2, CR3, and CR4 were identified in 2-DE gel with different *PI* values, but they matched the same amino acid sequence. It is known that a large number of isoforms are caused by single-nucleotide polymorphisms or SNPs, small genetic differences between alleles of the same gene. Currently, we cannot determine whether these isoforms are physiologically relevant, but the existence of multiple isoforms opens new areas for understanding gene functions in dinoflagellates.

**Figure 7:**
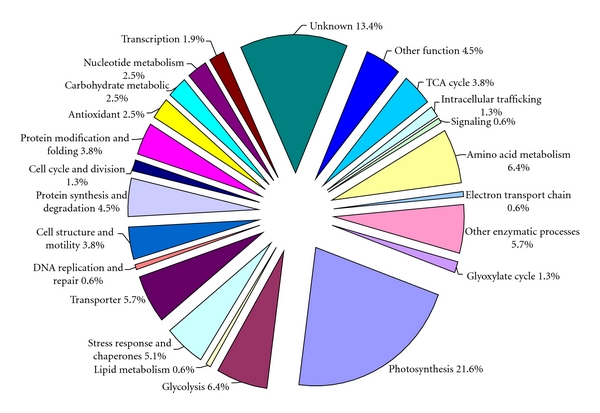
GO functional classification of the proteins identified in* A. tamarense.* The functional categories were defined according to Taylor and Johnson [41].

Based on the functional categories established [28], 158 unique proteins were classified into 23 groups (Figure 7). Among the unique proteins identified, 21.6% were involved in photosynthesis, 6.4% were in glycolysis, 6.4% in amino acid metabolism, 5.7% in other enzymatic processes, 5.7% were transporters, and 5.1% were involved in stress response or as chaperones. Other proteins, accounting for small number of the total, were related to protein synthesis and degradation (4.5%), cell structure and motility (3.8%), the TCA cycle (3.8%), protein modification and folding (3.8%), antioxidant activities (2.5%), carbohydrate metabolism (2.5%), nucleotide metabolism (2.5%), transcription (1.9%), the glyoxylate cycle (1.3%), the cell cycle and division (1.3%), intracellular trafficking (1.3%), DNA replication and repair (0.6%), lipid metabolism (0.6%), the electron transport chain (0.6%) and signaling (0.6%). Other functional and unknown function proteins accounted for 4.5% and 13.4% of the total protein, respectively.

## 4. Discussion

### 4.1. Protein Identification Strategy for Genome-Unsequenced Dinoflagellates

Dinoflagellates are not only the major causative agents of worldwide HABs but also are the producers of various potent biotoxin. However, a worldwide lack of available genetic information limits our understanding of HABs and consequently our ability to monitor, mitigate and prevent them. Proteomics provides effective strategies and tools for profiling and identifying dinoflagellate proteins in order to elucidate the biochemical and molecular mechanisms of bloom formation and toxin biosynthesis. Contemporary proteomics requires prompt and confident protein identification of proteins of interest. A sequence similarity search is a powerful tool for the identification of proteins from organisms with unsequenced genomes [33, 42–46]. In the past few years, various sequence similarity search engines, such as MS-BLAST [33], FASTS [43], CIDentify [41], MS-Homology [47], and OpenSea [48], have been developed and successfully applied in various proteomic studies. Partial sequence tags or complete peptide sequences were deduced directly from MS/MS spectra with no recourse to database resources [49] and then searched against a database in an error-tolerant fashion. In this way, even proteins with only marginal sequence similarity to reference database entries could be identified [42, 45, 46]. Recently, a layered manner combining LS-MS/MS analysis with stringent data processing and sequence similarity database search was developed and successfully applied to identify proteins in organisms with unsequenced genomes [34].

*De novo* sequencing analysis is a newly developed strategy for protein identification from incomplete- or nongenome organisms, which is regarded as the only alternative choice for the study of organisms with incomplete databases or databases not included in the public domain [20, 50–52]. This approach has been successfully applied in recent studies with incomplete- or nongenome organisms in order to characterize their proteins [19–23]. In this way, partial or complete amino acid sequences are obtained using either manual or automated *de novo* peptide sequence analysis. Manual protein sequencing can yield exact amino acid sequences without ambiguity via Edman degradation, but this procedure is time consuming and laborious. Moreover, its sensitivity is lower than mass spectrometry, and it is halted by the presence of blocked amino acids. Several automated software tools have been developed to deduce the amino acid sequences from an MS/MS spectrum [53–55], which consists of a ladder of peaks for *y-ions *(ions containing a *C*-terminus) and *b-ions *(ions containing an *N*-terminus). Interpretation of MS/MS spectra relies on calculating the mass differences between adjacent fragment ion peaks of *y*-series or *b*-series, which are common in tryptic peptides. *De novo *sequencing enables the analysis of quality MS/MS spectra which fail to generate protein identifications after database searches, which is the case for the majority of dinoflagellate proteins. 

In the present study, a multilayer, stringent and sequence similarity database searching strategy combining MALDI-TOF-TOF MS with *de novo* sequence analysis and stringent homology-based searching tools was developed, which provided a rapid and reliable means to identify proteins in *A. tamarense* with an unsequenced database. This data interpretation pipeline has no need for chemical derivatization or isotopic labeling of analyzed peptides or for repetitive MALDI-FOF-TOF analysis under specific settings, and is applicable to all two dimensional gel-based proteomic approaches for studying dinoflagellates. Moreover, it might also have important implications for proteomics in fully sequenced organisms, as it validates borderline hits produced by conventional database searches and has the potential for unbiased screening for PTMs, sequence polymorphism and unrecognized splicing variants.

### 4.2. Protein Functions of Dinoflagellates

*A. tamarense* is an autotrophic microalgae which uses CO_2_ and light as carbon and light sources. This study identified various light-harvesting proteins, chloroplast light-harvesting complex proteins, chl *a-* or *c*-binding proteins, and peridinin-chl *a*-binding proteins, which have been reported in many dinoflagellate species at the transcriptional level [27]. RuBisCO is the most abundant protein on earth and triggers reactions to make the carbohydrates, proteins, and fats used to sustain all forms of life. In our study, four isoforms of RuBisCO (spots CR1, CR2, CR3, and CR4) were identified abundantly in *A. tamarense*. Beside these isoforms, RuBisCO large subunits were also found in *A. tamarense*. RuBisCO has also been found widely in many dinoflagellate species. Moreover, several other proteins involved in the Calvin cycle, that is, chloroplast transketolase, ribulose-5-phosphate 3-epimerase, chloroplast phosphoribulokinase, ribulose bisphosphate carboxylase were also identified in *A. tamarense* these proteins are involved in various processes of the Calvin cycle and participate in carbon fixation. Glyceraldehyde-3-phosphate dehydrogenase (GAPDH) was another major component of the proteins identified. Nine spots (spots 51, 60, 63, 68, 71, 82, 85, 86, and 146) were identified as GAPDH, and they presented different cellular locations in *A. tamarense*. GAPDH is an enzyme that catalyzes the sixth step of glycolysis and thus serves to break down glucose for energy and carbon molecules. In addition to this function, GAPDH has recently been implicated in several nonmetabolic processes, including transcription activation, initiation of apoptosis and ER to Golgi vesicle shuttling. Sequences coding for this enzyme has also been reported amongst the highest expressed in the EST libraries of other dinoflagellates such as *A. catenella* [27], *L. polyedrum* [56], *A. tamarense* [26], *K. brevis* [57], and *A. fundyense* [58]. Another transferase enzyme, chloroplast phosphoglycerate kinase involved in glycolysis, was identified. It transfers a phosphate group from 1,3-biphosphoglycerate to ADP, forming ATP and 3-phosphoglycerate. Beside these proteins, a number of proteins involved in the light phase of photosynthesis, such as chloroplast ferredoxin-NADP (+) reductase, photosystems I subunit VII, cytochrome b6, PsbV, and chloroplast ATP synthase gamma-subunit, were identified in *A. tamarense*. Two proteins involved in chlorophyll synthesis, geranylgeranyl reductase, and plastid fructose-1,6-bisphosphate aldolase class II protein precursor were also identified.

Protein synthesis is a complex biological process, including amino acid elongation, protein folding, posttranslational modification, and protein degradation. Our study identified translational initiation inhibitor, peptidase, ribosomal protein, elongation factor, calretulin, protease, proteasome, and other protein-synthesis-related proteins in *A. tamarense*. These proteins participate in amino acid elongation, protein modification, folding, and degradation in *A. tamarense* cells. Moreover, two proteins (signal peptidase I and ADP-ribosylation factor-like 2) involved in intracellular trafficking were also identified in *A. tamarense*. These two proteins participate in the proteolytic processing of proteins or folding of tubulin peptides.

Seven proteins involved in amino acid metabolism were identified in *A. tamarense*, that is, methionine S-adenosyl transferase, S-adenosyl-homocysteine hydrolase-like protein, adenylyl sulfate kinase, glutamine synthetase, glutamate semialdehyde synthase, adenosylhomocysteinase, and ketol-acid reductoisomerase. These proteins participate in the biosynthesis and conversion of various amino acids in dinoflagellate cells.

Glycolysis is thought to be the archetype of a universal metabolic pathway that converts glucose C6H12O6, into pyruvate, CH3COCOO^−^ and H^+^. The free energy released in this process is used to form the high-energy compounds ATP and NADH. It occurs, with variations, in nearly all organisms, both aerobic and anaerobic. In this study, six proteins involved in different steps of glycolysis were identified; they were enolase, fructose bisphosphate aldolase, GAPDH, phosphoglucomutase, phosphoglycerate kinase, and triose-phosphate isomerase. 

Four proteins, peptidoglycan interpeptide bridge formation enzyme, alcohol dehydrogenase GroES domain protein, glucose-methanol-choline oxidoreductase, and a predicted protein were identified in *A. tamarense*. These proteins might be involved in cell wall formation, peptidoglycan synthesis, as glucose oxidase, and other functions.

In eukaryotic cells, the citric acid cycle (TCA) is part of a metabolic pathway involved in the chemical conversion of carbohydrates, fats, and proteins into carbon dioxide and water to generate a form of usable energy. Our study identified six proteins involved in the TCA cycle, that is, malate dehydrogenase, and its precursor, dihydrolipoamide acetyltransferase, isocitrate dehydrogenase and two hypothetical proteins. Furthermore, two proteins, phosphoglycolate phosphatase precursor and isocitrate lyase, involved in the glyoxylate cycle, were also identified, and these two proteins participate in glyoxylate and dicarboxylate metabolism.

Five ATPase regulating cation and calcium transports were identified in *A. tamarense*. ATPases are a class of enzymes that catalyze the decomposition of adenosine triphosphate (ATP) into adenosine diphosphate (ADP) and a free phosphate ion. This dephosphorylation reaction releases energy, which the enzyme (in most cases) is harnessed to drive other chemical reactions that would not otherwise occur. Some such enzymes are transmembrane ATPases which move solutes across the membrane, typically against their concentration gradient. Three other hypothetical transport proteins were also identified in our study, but their functions were not well known.

Little is known concerning the cell cycle regulation of dinoflagellate cells although a few cyclin-like proteins have been found in some dinoflagellate species. Our study identified two cell cycle regulating proteins, cell division protein FtsZ, and DNA damage checkpoint protein rad24. The former is the key protein in cell division while the latter is essential for DNA damage checkpoint control. Another cell cycle regulation protein, DNA polymerase, was also identified in this study which plays an important role in DNA replication and repair in eukaryotes.

Three transcriptional proteins, pseudouridine synthase, ATP-dependent helicase, and hypoxia-inducible factor 1 alpha inhibitor were identified from *A. tamarense*. These proteins play critical roles in maintaining the structure and integrity of DNA or RNA.

*A. tamarense* is a motile organism with two flagella which propel the cells through the water. In our study, actin, tubulin, and flagellin were identified from *A. tamarense*. Actin and tubulin being two major components of flagella and cilia in protists including dinoflagellates, while flagellin is a protein forming the filament in the bacterial flagellum. The presence of theses proteins indicated that they play important roles in the cell structure and motility of *A. tamarense*.

Stress proteins and antioxidant enzymes have been identified in dinoflagellate species [59]. In our study, two antioxidative enzymes, copper/zinc superoxide dismutase and superoxide dismutase, and two antioxidant proteins, peroxiredoxin V protein and a conserved hypothetical protein, were identified in *A. tamarense*. Heat shock proteins (HSPs) are highly regulated proteins that are involved in normal cellular activity and are upregulated when the cell is exposed to stress such as heat or excess ROS production. This study identified three HSPs, HSP60, 70 and 90, and one HSP chaperone, GroEL-like chaperone, ATPase in *A. tamarense*. A previous study demonstrates HSP 60, together with Mn SOD and Fe SOD in a dinoflagellate species, *Karenia brevis*, and these play an important role in the survival of this species.

Beside the above functional groups, numerous proteins involved in transcription, the electron transport chain, nucleotide metabolism, signaling, and lipid metabolism together with some other functional proteins were also identified from *A. tamarense*. It should be emphasized that most of the proteins identified in the present study have been predicted at transcriptional levels in various dinoflagellates [60, 61], which further demonstrated that the protein identifying method developed in this study was rapid and reliable, although some proteins were identified with unknown functions. In future, more effort should be devoted to both transcriptomic and genomic studies of dinoflagellates, which will facilitate protein identification, and to proteomic studies which will aid in gaining an understanding of HABs and the subsequent monitoring, mitigation, and prevention of HABs.

In summary, the current study was undertaken to delineate a proteomics scale methodology to identify proteins from dinoflagellates. Using this methodology, 116 out of the 220 excised protein spots, representing high, moderate, and low abundant proteins, gave positive identification. Most of them have been predicted at the transcriptional level or have been identified from various dinoflagellate species and play important roles in the various physiological activities of dinoflagellates. Nevertheless, the present results provided the first preliminary proteomic profile and 2-DE gel reference map of *A. tamarense* and will form the basis of future proteomics scale studies using the unsequenced database of *A. tamarense*.

## 5. Supporting Information

List of all peptide sequences deduced from each MS/MS spectrum using DeNovo Explorer software *de novo* sequencing.

## Supplementary Material

Supplementary material is a complete list of proteins identified using layered identification method includes the protein name, accession number, biological functional term and threshold value.
